# Annotations

**Published:** 1892-11-05

**Authors:** 


### Annotations.

**Converted Clowns as Hospital Chaplains.**

The *Citizen* has made itself merry over the conduct of the Metropolitan Asylums Board, which tried, but failed, to agree about the appointment of a new chaplain for the North-Eastern Hospital last week. The chaplain in question is the Rev. R. B. Raffles, M.A., and the amount of his salary is £100 per year. Some of the managers, who probably have an objection to the deliberate starving of a clergyman, suggested that the £100 a year might be supplemented by board and lodging. But this was too much for the virtuously economical souls of some of the members of the Board. Why did the North-Eastern Hospital want a chaplain at all? asked one of these gentlemen, seeing that a great many of the inmates were children. But this adverse criticism was met by the suggestion that a resident chaplain would be able to amuse the children, and to see that they were well employed throughout the day. Whereupon the *Citizen* wittily suggests that a converted clown at £50 a year would be the most suitable sort of chaplain to meet the objections of the economists, and to fulfil the duties of the position. It is to be hoped that this ludicrous way of putting the thing will not be without its effect upon the cast-iron minds of some of the members of the Metropolitan Asylums Board. With regard to the general question of religious ministrations to hospital inmates, there is no doubt whatever that every considerable hospital ough to have its chaplain, and in hospitals where it is practicable he should reside on the premises. Moreover—and we feel strongly on this point—he should be properly paid. To offer a gentleman with an university degree, and with a character which fits him for the office of chaplain, such a sum as £100 a year is not merely scandalous, it is senseless. For a rich country like our own to attempt to pay its public servants in this way is too much for one's patience. If Mr. Raffles is worthy of the appointment of chaplain, he is worthy of reasonable payment; and to offer him the starvation pittance of £100 a year is a proceeding of which the Metropolitan Asylums Board ought to be thoroughly ashamed. The truth is, that these gentlemen sometimes forget to be gentlemen when they are assembled in their corporate capacity.

**Heart Disease.**

Nothing better shows the real and intelligent progress of medical science during the last half century than the comprehensive and rational treatment of diseases of the heart. The *Practitioner* for October, in a short article of a page and a half, sets forth certain general principles which those medical men, who have no time for study, might with great advantage commit to memory, and which sufferers from heart affections should themselves be constantly taught by their attendant physicians. Twenty years ago, if a man was known or believed to have disease of the heart, he was as certain to be plied with digitalis as a hand-fed baby with the bottle. "Heart"—"digitalis"—the one idea connoted the other, in many medical minds, with a uniformity which was absolute. Nothing could be more absurd. Just as well might we associate together with unvarying regularity "stomach" and "rhubarb" or "bowel" and "castor oil." Digitalis is an admirable remedy, as everyone knows who has used it intelligently and in sufficient doses. But to force the pumping heart to work ever harder and harder, when rest is the one thing it needs, and to try to make it overcome obstructions in the circulation when the obstructions can be much better overcome by other means, is one of the speediest, but also one of the most distressing methods of killing a patient which even medical science has discovered. Medical men of limited experience in heart cases—and there are many such—should buy the October number of the *Practitioner*, if for no other purpose but to read and study the article on "The Treatment of Valvular-Diseases of the Heart."

**Medical Peers.**

Is it correct to speak of that which does not exist? But is it true that medical peers do not exist? They certainly exist in fact, if not in name. There are some medical men now living who are the "peers" of the highest and the worthiest in every rank of life. "For services rendered" is the label which the State ought to attach to every individual it selects from among his fellows and elevates to permanent rank. That was the label which everybody in the land affixed for himself to doughty and unconquerable Wellington. Lord Tennyson's peerage was acclaimed with a like unanimous accord. Life saving and health giving is a work of some importance in the land. If the soldier defends his country in the mass, the doctor defends its people in detail. The work in each case is the same. The great doctor is really a great man, believe it or not. He cannot be otherwise. The members of the medical profession who are thinkers know well what a vast amount of intellectual work and worthiness goes to the making of a really great doctor. The thirty thousand doctors of this country would like to see one or two medical peers. They are too proud to complain, and they know the worth of their own work too well to feel humiliated. But in their heart of hearts they think their fellow-countrymen a little unjust in this matter. Many of us console ourselves with the thought that non-medical people have not enough of scientific knowledge to enable them to appreciate properly the value of the work of medical science. We feel assured that as education advances this adverse balance will be redressed. In the meantime, however, her Majesty the Queen is a woman of great intellectual endowments; Mr. Gladstone is a man of scientific capacity, and the press of the country knows very well what really able men we have at the head of the medical profession. These circumstances being so, we feel that the due recognition of medical eminence ought not to hang fire any longer. It was only a few weeks ago that Mr. Gladstone reiterated an old opinion of his to the effect that medical men are destined to play an important part in the leadership of the future. He knows well, by many years of personal experience, how competent, zealous, and devoted to duty medicine can be at its best. There are several medical men whom we should like to see in the peerage, notably Sir William Jenner, Sir James Paget, and Sir Andrew Clark. Sir William Jenner, it is whispered, does not desire the honour; he feels that at his age it would be a name and nothing more. Sir James Paget, too, is well on in the seventies, though he is hale and robust. Sir Andrew Clark is still only about midway in the sixties, and is, intellectually, at his best, and physically, very little the worse for wear. If he were called to the upper House he could render the greatest possible services to the State. Medicine, as Mr. Gladstone has said, is destined to play a leading part in the future, and we are certain that the sooner this is generally recognised the better it will be for the general wellbeing. The general world is a good deal behind the doctors in all kinds of practical science that make for health, pleasure, and prosperity. Mr. Gladstone, we venture to think, would not only please an important profession, but bring about—as is much to be desired—the fulfilment of his own prophecy if he were to recommend one or two medical names to the Queen for the honour and dignity of the peerage.

